# Extrusion processing of peanut vine-corn-urea mixture promotes the growth performance of fattening cattle by modulating rumen fermentation and microbiota

**DOI:** 10.5713/ab.250978

**Published:** 2026-04-16

**Authors:** Xiaohui Kong, Mingyan Wang, Binghan Dong, Haoliang Tian, Di Ding, Tengyun Gao, Tong Fu, Chuanyou Su, Liyang Zhang

**Affiliations:** 1 Henan International Joint Laboratory of Nutrition Regulation and Ecological Raising of Domestic Animal, College of Animal Science and Technology, Henan Agricultural University, Zhengzhou, China; 2 Department of Economic Management and Animal Husbandry, Ruzhou Vocational and Technical College, Pingdingshan, China

**Keywords:** Diet Digestibility, Effective Degradability, Non-protein Nitrogen, Nutrient Composition, Peanut Vine

## Abstract

**Objective:**

This study aimed to develop a novel extruded compound feed, characterize its rumen degradation parameters, and investigate the effects on growth performance, diet digestibility, rumen fermentation, and bacterial community of fattening cattle.

**Methods:**

To determine the optimal ratio, we measured key nutritional components and ruminal degradability using *in situ* nylon bag technology. Subsequently, 36 fattening cattle (618.0±23.3 kg) were randomly assigned to the control group (CON) and the extruded compound feed group (EXP), with 18 cattle per group. The feeding experiment lasted 60 d.

**Results:**

With the increase in corn percentage in the extruded compound feed, the effective degradability of neutral detergent fiber (NDF) increased (p = 0.056). At the ratio of peanut vine: corn: urea of 65:30:5, the effective degradability of NDF reached the highest value of 27.3%. This extruded compound feed was used in the subsequent feeding experiment. The EXP group had higher average daily feed intake (CON vs. EXP: 16.0 kg vs. 17.2 kg, respectively, p<0.001) and average daily gain (CON vs. EXP: 1.51 kg vs. 1.72 kg, respectively, p<0.001). The feeding profit per head of fattening cattle in the EXP group was 7.1 US dollars higher than that in the CON group. The 16S rRNA sequencing results showed that the relative abundance of Bacteroidetes (CON vs. EXP: 37.7% vs. 59.2%, respectively, p<0.001) and *Prevotella*_1 (CON vs. EXP: 16.0% vs. 36.1%, respectively, p<0.001) in the rumen of cattle fed the extruded compound feed was increased.

**Conclusion:**

These results revealed that the optimal ratio of peanut vine: corn: urea was 65:30:5, representing the most efficient and cost-effective composition. The extruded compound feed is a promising feedstuff, that can significantly enhance rumen fermentation and growth performance in fattening cattle.

## INTRODUCTION

The feeding of fattening cattle plays an indispensable role in providing high-quality beef products for the global population [1]. Given this trajectory, it is crucial to improve production efficiency and feeding effectiveness in the fattening cattle feeding industry. Fattening cattle are in a stage of rapid weight gain and high feed utilization, during which they need to supplement a large amount of nutrients to meet its weight gain requirements [2]. Peanut vine are a typical byproduct of peanuts, which could be used as a high-quality source of roughage due to its certain nutritious value and soft texture. However, the high crude fiber content limits its application in production. Enhancing the nutritional value of feed through appropriate feed combinations during the production process might also be an economically effective method for the efficient feeding of fattening cattle.

A large number of studies have shown that extrusion processing could destroy plant cell walls and anti-nutritional molecules, thereby promoting starch degradation [3,4]. Pasqualone et al [5] noted that the palatability and storage properties of extruded straw feed could be enhanced. However, as a roughage resource, peanut vine has low starch content and is poorly suited for extrusion alone. Corn and urea are significant sources of energy and non-protein nitrogen (NPN) for ruminants. Zhu et al [6] pointed out that animals fed extruded corn exhibited not only greater ruminal degradability of dry matter (DM) and starch but also improved growth performance. The rapid decomposition of starch for energy could synchronize the rate at which rumen urea breaks down to create ammonia and microbial proteins. Based on the principle of rumen nitrogen balance, adding urea to grain and hay feed for fattening cattle could improve production performance. Alrez et al [7] reported that dietary urea supplementation improved nitrogen availability, enhancing ruminal microbial activity and overall diet digestibility. Ma et al [8] have indicated that there was an interactive effect between feeds, and a suitable combination of feeds could produce a positive feedback effect, thereby improving the utilization efficiency of feed.

The continuously increasing starch, NPN, and bioactive substances might serve as nutritional supplements in fattening cattle feed to promote rumen degradation and improve rumen fermentation. We assumed that supplementation with extruded compound feed could meet the nutritional requirements of fattening cattle by providing digestible fiber, energy, and NPN. Consequently, this study aimed to evaluate the effects of extruded compound feed on growth performance, diet digestibility, rumen fermentation parameters, and bacterial community of fattening cattle.

## MATERIALS AND METHODS

### Processing of extruded compound feed

The peanut vine and corn used in this experiment were harvested in the plantation area of Henan Province (central region of China). The extruded compound feed was formulated with different proportions of raw materials including peanut vine, corn and urea. The content of corn gradually increases to optimize the extrusion effect. The compatibility proportions of peanut vine: corn: urea were as follows (DM basis %): 60:35:5, 65:30:5, 70:25:5 and 75:20:5 respectively.

Then, the mixed feeds were extruded using an extruder (EXP160S-110Kw; Zhengzhou Doler Machinery) according to the manufacturer’s parameters (Henan Shennong Extruded Feed Technology). The extrusion duration was 5–15 s to produce the required extruded compound feed.

### Laboratory analysis of nutritional components

The feed samples were dried to constant weight in an oven at 55°C for DM determination, ground with a chopper, and passed through a 1-mm sieve for chemical analysis. The content of crude protein (CP) was determined by the Kjeldahl definition nitrogen method. The neutral detergent fiber (NDF) and acid detergent fiber (ADF) contents were determined according to a previous study, using a heat-stable α-amylase and sodium sulfite [9]. The ash content was measured through complete combustion at 550°C for 5 h. The calcium (Ca) content was determined through atomic absorption spectrophotometry, while the phosphorus (P) content was determined through colorimetry.

### *In situ* ruminal degradability

#### Animals, management, experimental design

Three healthy cattle with permanent rumen fistulas were used for measuring the ruminal degradability. The cattle were kept alone in the corral, drinking water freely, and fed a total mixed ration (TMR) at 8:00 am and 6:00 pm every day. The concentrated feed ingredients were corn silage, wheat straw, peanut vine, corn, wheat bran, soybean meal, distillers dried grains with solubles, cottonseed meal, vitamin-mineral premix, and sodium bicarbonate. The nutritional components are as follows (DM basis): NE_mf_ 5.43%, CP 11.45%, Ash 5.12%, NDF 38.18%, ADF 21.60%, Ca 0.52%, and P 0.24%.

After the samples were dried and pulverized, part of the samples were used for the determination of feed nutritional components, while the other part was sieved through a 2-mm sieve for rumen degradation experiments. Nutritional components of the sample were determined according to the above description.

#### Ruminal degradability

The experiment follows the principle of “simultaneously put and sequentially take out”. The 6.0 g sample was accurately weighed and put into a new nylon bag with a diameter of 50 μm and a specification of 8 cm×12 cm. Every 4 nylon bags were fixed with rubber bands at the crack of 1 PVC plastic tube (2 extruded, 2 unextruded). The plastic tubes were placed into the rumen of 3 experimental cattle at the same time point (1 h before morning feeding). The incubation time was set for 0, 4, 8, 12, 24, 36, 48, and 72 h. Each dietary sample was repeated in triplicate. At each time stage, the nylon bags were removed from the rumen and slowly rinsed with water until they were clean. The rinsed nylon bag samples were dried to constant weight at 65°C, and then the feed samples were crushed to determine the contents of DM, CP, NDF, and ADF. The degradation parameters and effective ruminal degradability (ED) were calculated using the formula provided by Ørskov and McDonald [10].


(1) $$\text{P}=\text{a}+\text{b}\times (1-{\text{e}}^{-\text{ct}})$$

(2) ED=a+([b×c]/[c+k])

Where P is the ruminal disappearance at time t (%), a is the soluble fraction (%), b is the slowly degradation fraction (%), c is the slowly degradation rate (%), and k is the estimated ruminal flow rate (%/h).

### Feeding experiment

Thirty-six Holstein fattening cattle with similar body weight (BW) (618.0±23.3 kg) were randomly divided into 2 groups, with 18 cattle in each group. The diets were designed to be isonitrogenous and isoenergetic with the same source of feed raw materials, as follows: 1) the corn-soybean meal-peanut vine-corn silage-based diet (CON, used as the control diet), 2) the extruded compound feed (peanut vine: corn: urea = 65:30:5) was used to substitute raw materials in equal proportions (EXP). The diet was formulated based on recommendations provided by the National Research Council NRC [11] to satisfy the nutritional needs of fattening cattle.

The experiment lasted 74 d in total, consisting of a 14 d adaptation period followed by a 60 d formal feeding period for sample collection. During the feeding period, the fattening cattle were kept individually in pens. The experimental diet was fed at 8:00 a.m. and 6:00 p.m., respectively. Adjust daily feed quantities to maintain a residual amount between 100–150 g of the total provision. The ingredients and nutrient composition of the diet are shown in Table 1.

#### Intake and growth performance

Throughout the entire experiment, the starting feed intake of each individual was calculated as average daily feed intake (ADFI) by subtracting the rejection amount from the provided amount. The BW was recorded before early feeding on days 0, 30, and 60. The average daily gain (ADG) and feed-to-gain ratio (F/G) were measured using the previous measurements.

#### Economic benefits

During the trial period, the feed unit price was calculated based on the raw material prices and formula proportions of each experimental diet. The feeding profit was estimated in combination with the market price of fattening cattle. The total feed cost and weight gain income for each group were determined according to the ADFI and total weight gain of the fattening cattle.


(3) Total feed cost=ADFI×Feed unit price×Feeding days

(4) Weight gain income=Total weight gain×Unit price of fattening cattle

(5) Feeding profit=Weight gain income-Total feed cost

#### Diet digestibility

The endogenous indicator method was used to assess *in vivo* digestion immediately following the end of the formal feeding period. Eighteen cattle (9 cattle/group) were selected according to BW and transferred to separate corrals. Fecal samples (approximately 100 mL) were collected twice daily at 08:00 and 15:00 for 3 consecutive days, with stringent hygiene protocols maintained throughout the collection period to minimize sample contamination. The collected fecal samples were dried at 55°C for 48 h and then ground in a grinder until passed through a 1-mm sieve to make oven-dried samples, which were stored in ziplocked bags. The contents of DM, CP, ether extract (EE), NDF, and ADF in fecal samples were determined by the above description. Nutrient digestibility estimations were all based on each animal’s DM intake and orts [12].

#### Ruminal fermentation parameters

Rumen fluid samples were collected from each cattle at the end of feeding trial (18 cattle/group). According to the procedure of Zhang et al [13], rumen fluid samples (30 mL) were obtained from each fattening cattle through a flexible stomach tube with a thickness of 2-mm and an inner diameter of 6-mm. The samples were filtered on 4 layers of sterilized gauze to remove impurities and preserved in liquid nitrogen. The follow-up assay consisted of three parts: one part of rumen fluid was used to determine pH and volatile fatty acids (VFAs) concentrations, the second part was used for ammonia nitrogen (NH_3_-N) concentration analysis, and the third part was used for DNA extraction and rumen microbiota analysis. Rumen fluid pH was determined using a PHB-4 portable pH meter (Shanghai Yi Electrical Scientific Instrument).

#### Ruminal bacterial community composition

Twelve rumen fluid samples (6 samples/group) were randomly selected for DNA extraction and ruminal bacterial community analysis. Firstly, using a DNA kit (Qiagen), according to the manufacturer’s protocols. The V3 to V4 hypervariable region of the bacterial 16S rRNA gene was amplified using bacterial primer pairs (5′-ACTCCTACGGGAGGCAGCA-3′ and 5′-GGAC TACHVGGGTWTCTAAT-3′) [14]. The integrity of the PCR product was verified, purified, and quantified, and then its concentration was measured. Subsequently, the 16S rRNA gene amplicons were sequenced using the Illumina HiSeq 2500 system (Illumina). Sequences that shared greater than 97% similarity were clustered into a single operational taxonomic unit (OTU). The highest abundance sequence in each OTU was selected as its representative, and then taxonomically annotated using the RDP classifier against the GreenGene database. MOTHUR v1.3.0 software was used to calculate the sparse curve and alpha-diversity index.

### Statistical analysis

The data were analyzed using SAS/STAT software (ver. 9.2; SAS Institute). All data were tested for normality using the UNIVARIATE procedure. Nonlinear regression was performed using the NLIN procedure on rumen degradation data at different incubation time points. For growth performance data, a MIXED model was employed, and the statistical model was explicitly defined as: *Y_ijk_* = *μ*+*T_i_*+*W_j_*+*T_i_×W_j_*+*B_k_*+*ɛ_ijk_*, where *Y_ijk_* is the observed value of the growth performance index for the *k*-th experimental unit under the *i*-th treatment at the *j*-th time point, *μ* is the overall mean, *T_i_* is the fixed effect of the *i*-th treatment, *W_j_* is the fixed effect of the *j*-th time point, *T_i_×W_j_* is the fixed interaction effect between treatment and time point, *B_k_* is the random effect of the *k*-th experimental unit (individual fattening cattle), and *ɛ_ijk_* is the random residual error.

Statistical significance of differences among rumen degradation parameters was tested using the ANOVA procedure, and the statistical model was explicitly defined as: *X_ij_* = *μ*+*α_i_*+*ɛ_ij_*, where *X_ij_* is the *j*-th observation under the *i*-th treatment group, *μ* is the overall mean, *α_i_* is the fixed effect of the *i*-th treatment (*i* = 1, 2, ..., *k*), *ɛ_ij_* is the random experimental error associated with the *j*-th observation in the *i*-th group. The apparent digestibility of nutrients, rumen fermentation parameters, and rumen bacterial community were compared using Tukey’s test. Data were presented as least squares means and their standard errors of the mean (SEM). Significance was declared when p<0.05.

## RESULTS

### Chemical composition of peanut vine compound feed

As shown in Table 2, the results indicated that as the proportion of peanut vines in extruded compound feeds increased, CP concentrations increased (p<0.05), while NDF and ADF concentrations decreased (p<0.05). The extrusion processing reduced the NDF and ADF contents of all peanut vine compound feed (p<0.05).

### *In situ* effective degradability

The parameter estimates of ruminal degradability are shown in Table 3. The extrusion processing increased soluble fraction and effective degradation of DM in all peanut vine compound feeds (p<0.05). As the corn content in the extruded compound feed increased, the effective degradability of NDF increased (p<0.05), with the highest being achieved when the proportions of peanut vine: corn: urea was 65:30:5. This formulation of extruded compound feed was also used as the EXP diet in a subsequent beef cattle feeding experiment.

### Intake and growth performance

The feed intake and growth performance results of fattening cattle are shown in Table 4. During the entire experimental period, compared with the CON group, the ADFI and ADG of fattening cattle in the EXP group were increased (p<0.001). With the increase of feeding period, both ADFI and ADG increased (p<0.05), and an interaction effect on ADFI was observed (p<0.001).

### Economic benefits

As shown in Table 5, compared with the CON group, the feed cost per head of fattening cattle in the EXP group increased, while the total weight gain was 12.6 kg higher. From the perspective of feeding profit of fattening cattle, the EXP group achieved a gross profit of 7.1 US dollars (USD) per head higher than the CON group.

### Diet digestibility

The results of the diet digestibility are shown in Table 6. The diet digestibility of DM, CP, and NDF in the EXP cattle was higher than the CON cattle (p<0.05).

### Ruminal fermentation parameters

The results of the ruminal fermentation parameters are shown in Table 7. The results showed that the addition of extruded compound feed to the cattle diet reduced rumen pH value (p<0.001). The concentrations of NH_3_-N, acetate, propionate, total volatile fatty acids (T-VFA), and the acetate-to-propionate ratio in the rumen of cattle fed the extruded compound feed were higher than those in the CON cattle (p<0.05).

### Ruminal bacterial community composition

**Figure 1:**
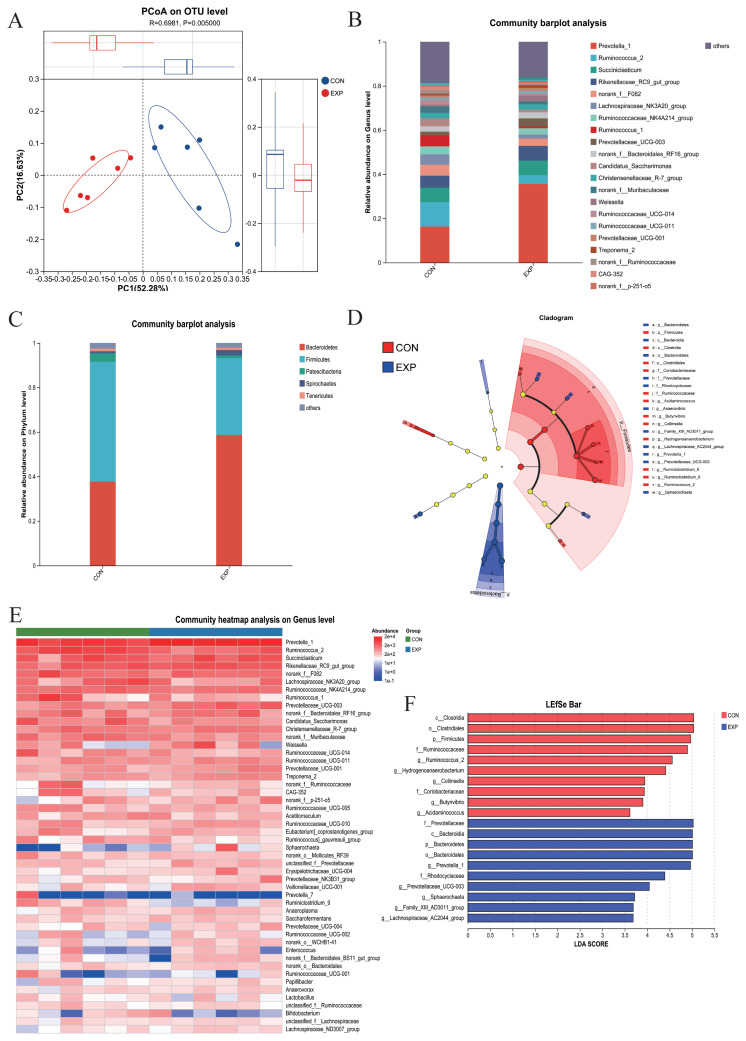
Effects of feeding extruded compound feed on rumen bacterial community composition. (A) The relative abundance of rumen bacterial at the phylum level in two groups of fattening cattle. (B) The relative abundance of rumen bacterial at genus level in two groups of fattening cattle. (C) The PCoA analysis of rumen bacterial in two groups of fattening cattle. (D) Heat map showed the relative abundance of rumen bacterial in the two groups at the genus level. (E) Phylogenetic cladistics of different species of rumen bacterial in two groups of fattening cattle. (F) Distribution map of LDA value of different species of rumen bacterial in two groups of fattening cattle. CON, the corn-soybean meal-peanut vine-corn silage-based diet (used as the control diet); EXP, the extruded compound feed (substituted equal amount of feed ingredients).

As shown in Table 8, the PCoA analysis revealed a difference in rumen bacterial clusters of cattle fed the extruded compound feed compared to the CON cattle (p<0.05, Figure 1A).

The principal rumen bacterial phylum in the CON and EXP cattle were Bacteroidetes (37.7%–59.2%), Firmicutes (53.9%–34.0%), and Patescibacteria (3.9%–1.2%), with relative abundance greater than 0.1% (Figure 1C). At the genus level, the most predominant bacteria in fattening cattle fed with the CON and EXP diets were *Prevotella*_1, accounting for 16.0% and 36.1% of all sequences, respectively, followed by *Ruminococcus*_2, *Suciniclasticum*, and others (Figure 1B and E). The LEfSe analysis on the differences in bacterial structure between the two groups showed that the bacteria with the most differences in the CON cattle were *Clostridia*, *Clostridiales*, and *Ruminococcaceae*, while the bacterial genera with the greatest differences in the EXP cattle were *Prevotellaceae*, *Bacteroidia*, and *Prevotella*_1 (Figure 1D and F).

## DISCUSSION

In this study, the extrusion process decreased the content of NDF and ADF in the peanut vine compound feed, which was consistent with the results reported by Broderick et al [15]. The ruminal degradability of nutrients is an important indicator for evaluating the nutritional value of feed and rumen microbes’ ability to convert feed. In the feeding of fattening cattle, the utilization of agricultural byproducts (such as peanut vine) as a diet could fully exploit the animal’s digestive potential and reduce feeding costs [16]. In this study, the ED of DM and CP in the extruded diets was higher than that in the unextruded diets. These results explained that the extrusion of peanut vine and corn in the diet under high temperatures and pressure could break the chemical bonds of plant fiber molecules, thus improve its degradation rate. The findings of this study were consistent with the He et al [17]. The internal environment of the rumen is an extremely complex microbial system, in which various microorganisms play different roles [18]. In this study, when the ratio of peanut vine, corn, and urea was 65:30:5, the ED of NDF in the diet was the highest, indicating that the extruded compound feed with this ratio had higher nutritional value.

Dietary nutrient composition and palatability are the most critical factors determining animal performance [19]. The ADFI and ADG of the EXP cattle were higher than the CON cattle from the perspective of the entire growth period, which was primarily due to the fact that after the peanut vine and corn were extruded, it could improve its palatability, promote intestinal peristalsis. Zhang et al [20] concluded that supplementing peanut vine to the diet of Holstein cattle could satisfy the animals’ growth needs and enhance the digestibility of its rumen. It is noteworthy that the lower F/G ratio in EXP cattle might be due to reduced fiber and lignin content after extrusion, enhancing ruminal degradation and overall feed utilization. This conclusion was congruent with the Zhang et al [21] research. It could be seen that feeding compound extruded peanut vine diet had a better effect on improving the growth performance of fattening cattle than the unextruded diet.

Feeding efficiency is an important indicator for evaluating the application value of feed ingredients in feeding production. Improving growth performance by optimizing dietary nutrient levels is an effective approach to enhance economic benefits. The results of this experiment indicated that supplementation of the extruded compound feed increased ADFI and total weight gain of fattening cattle. The feeding profit per head of fattening cattle in the EXP group was 7.1 USD higher than that in the CON group. This might be attributed to the improved palatability of the compound feed after extrusion treatment, which effectively enhanced growth performance and immune function of fattening cattle, thereby improving feeding efficiency.

The apparent digestibility of nutrients can directly reflect the digestibility of fattening cattle to the diet [22]. The results of this experiment showed that the apparent digestibility of DM, CP, NDF, and ADF in the EXP cattle were higher than the CON cattle. This might be due to the extrusion of peanut vine and corn, which met the requirements of fattening cattle for CP, NDF, and ADF, increased the activity of rumen microorganisms. However, there was no difference in the apparent digestibility of EE between the two groups, which might be related to the extrusion treatment and quality of peanut vine and corn. Wang et al [23] reported that the apparent digestibility of NDF could be improved after animals’ diets were extruded. Cueva et al [24] also showed that supplementing extruded grains to dairy cow diets could increase the apparent digestibility of DM and CP. On the one hand, extrusion induced protein denaturation and lignin breakdown might generate Maillard products, thus improving nutrient utilization. On the other hand, the degradation of fiber and lignin in ruminants was mainly based on the adhesion of rumen microorganisms [25,26]. According to the research of Bao et al [27], extrusion could destroy the dense structure of the cell wall, increase feed surface area, enhance rumen microorganism adhesion, and facilitate the hydrolysis of digestive enzymes, all of which improve the utilization rate and enzymatic hydrolysis efficiency. Therefore, the above results indicated that fattening cattle have higher digestibility and absorption of various nutrients when fed with an extruded compound feed.

The rumen pH value, VFA, and NH_3_-N concentration are crucial indicators to reflect rumen fermentation of feed. Hay-based diets could promote VFA production to achieve rumen development. Izadbakhsh et al [28] identified that increasing hay in the diet stimulated cellulolytic microorganisms and redirected rumen fermentation toward more VFA production. Omidi-Mirzaei et al [29] indicated that the concentration of T-VFA in the rumen increased linearly with the proportion of hay in the diet. In the current study, the rumen T-VFA concentration of fattening cattle with the EXP was the highest. This might be because peanut vine are rich in fiber and lignin, which increases the rumination duration of fattening cattle, thereby promoting the rumen to produce more VFA, consistent with the above research results. The rumen pH value is an essential indicator of microbial fermentation and the internal environment [30]. In this study, the rumen fluid pH of fattening cattle in each group was 6.47–6.86, all within the normal range, demonstrating that feeding hay had no effect on rumen pH and had a positive impact on maintaining homeostasis of the internal environment. The pH value of the CON cattle was higher than the EXP cattle, which might be related to the extrusion quality of the extruded compound feed and the different physiological conditions of the animals. Acetate and propionate are the primary sources of VFA [31]. In the diet, the EXP cattle had the highest NH_3_-N content for fattening cattle, which reflects the degree of degradation of microorganisms to nitrogen-containing substances in the substrate. This study revealed that after extruded treatment, peanut vine compound feed enhances animals’ chewing of feed, thus promoting saliva flow to the rumen and increasing the concentration of NH_3_-N in the fermentation fluid.

The diversity of ruminal bacterial communities is a key factor affecting rumen function [32]. The 16S rRNA sequencing revealed that there was no difference in the alpha-diversity of ruminal bacterial communities in fattening cattle fed with different diets. However, the Sobs, ACE, and Chao1 indices in the EXP cattle showed an increasing trend, illustrating that the supplementation of extruded compound feed to the diet had a subtle beneficial regulatory effect on the diversity of ruminal bacterial. The PCoA results revealed that there was obviously clustering separation of ruminal bacterial between the groups, implying that the two groups formed different ruminal bacterial communities.

At the phylum level, the increase of Bacteroidetes and the decrease of Firmicutes were observed in the rumen in the EXP cattle. The increasing proportion of Bacteroidetes and Firmicutes had a high correlation with improving the apparent digestibility of nutrients and promoting growth [33]. Consistently, the ADFI, ADG, and dietary apparent digestibility of fattening cattle were enhanced after supplementing the extruded compound feed to the diet. At the genus level, the relative abundance of *Prevotella*_1 and *Bacteroidia* of fattening cattle in the EXP group were increased. Previous research has demonstrated that *Prevotella*_1 could improve the decomposition of cellulose in the rumen, produce propionate by the fermentation of sugars through the acrylic and succinic acid pathway [34]. In current experiment, the content of propionate in the rumen of the EXP cattle was increased after feeding the extruded compound feed, which was consistent with this conclusion. The reason might be that the hemicelluloses and plant cell walls were degraded after the extrusion of peanut vine and enhanced the transformation of sugars, amino acids, and peptides, thus promoting the proliferation of *Prevotella*_1*. Bacteroidia* was the dominant bacteria in the intestinal. Its relative abundance was positively correlated with feed utilization and VFA metabolism, which was consistent with the increase of ADG and T-VFA of the EXP cattle.

## CONCLUSION

This study developed a new extruded compound feed formulated by extruding a mixture of peanut vine, corn, and urea, which was characterized by high levels of energy, NPN and higher digestible fiber. Under the feeding conditions of this study, the optimal proportion of peanut vine: corn: urea was 65:30:5, which resulted in the highest effective degradation rate of NDF and the best economic efficiency. The feeding experiment results showed that the diet improved the growth performance of fattening cattle by increasing the ADG and ADFI, and the feeding profit per head of fattening cattle was 7.1 USD higher than that of the CON group. It also improved nutrient utilization by regulating rumen fermentation, increasing T-VFA production, optimizing the acetate-to-propionate ratio, and promoting the enrichment of beneficial bacteria such as Bacteroidetes and *Prevotella*_1. This study provides a promising strategy for utilizing peanut vine agricultural byproducts in ruminant production.

## Figures and Tables

**Table 1:** The ingredients and nutrient composition of the diets in the feeding experiment (DM basis) %

Items	Treatments^1)^	

	CON	EXP
Ingredients (% of DM)		
Corn	28.0	16.0
Wheat bran	6.0	6.0
Soybean meal	10.0	10.0
Cottonseed meal	2.0	2.0
Urea	2.0	0
Whole plant corn silage	24.0	24.0
Extruded compound feed	0	40.0
Peanut vine	26.0	0
Vitamin-mineral premix^2)^	2.0	2.0
Total	100.00	100.00
Nutrient composition		
NE_mf_ (% of DM)^3)^	5.42	5.42
CP	17.34	17.59
EE	3.76	3.19
NDF	33.41	30.05
ADF	19.17	17.32
Ca	0.75	0.76
P	0.31	0.31

1) CON: the corn-soybean meal-peanut vine-corn silage-based diet (used as the control diet); EXP: the extruded compound feed (substituted equal amount of feed ingredients).

2) Vitamin-mineral premix contained (per kg of DM): 500,000 IU of vitamin A, 50,000 IU of vitamin D, 2,500 IU of vitamin E, 0.003–0.005 kg of sodium chloride, 2,490 mg of iron, 615 mg of copper, 2,001 mg of manganese, 1,668 mg of zinc, 500 IU of biotin.

3) Calculated from NRC [11], while the other values were measured.

DM, dry matter; CP, crude protein; EE, ether extract; NDF, neutral detergent fiber; ADF, acid detergent fiber; Ca, calcium; P, phosphorus.

**Table 2:** Comparison of nutrient composition in compound feed with different proportions before and after extruded (DM basis) %

Items	Treatments (peanut vine: corn: urea)								SEM	p-value

	60:35:5		65:30:5		70:25:5		75:20:5			

	Unextruded	Extruded	Unextruded	Extruded	Unextruded	Extruded	Unextruded	Extruded		
DM	0.92	0.91	0.91	0.92	0.92	0.92	0.92	0.92	0.002	0.991
CP	23.5^e^	23.8^de^	23.7^de^	23.9^cd^	24.1^bc^	24.3^ab^	24.4^ab^	24.5^a^	0.08	<0.001
NDF	30.7^cd^	25.8^f^	32.7^bc^	27.6^ef^	34.5^ab^	29.5^de^	36.0^a^	32.3^bcd^	0.85	<0.001
ADF	22.6^cd^	20.1^d^	24.6^bc^	22.3^cd^	26.6^ab^	24.2^bc^	28.4^a^	26.1^ab^	0.66	<0.001
Ash	4.16^c^	4.29^c^	4.45^bc^	4.38^bc^	4.79^abc^	4.78^abc^	5.16^a^	4.99^ab^	0.09	0.004

a–f Superscripted letters indicate significant differences based on Tukey’s tests, with significance defined as p<0.05.

SEM, standard errors of the mean; DM, dry matter; CP, crude protein; NDF, neutral detergent fiber; ADF, acid detergent fiber.

**Table 3:** Rumen degradation parameters of compound feeds with different proportions before and after extruded

Items^1)^	Compatibility proportion of peanut vine compound feed (peanut vine: corn: urea)								SEM	p-value

	60:35:5		65:30:5		70:25:5		75:20:5			

	Unextruded	Extruded	Unextruded	Extruded	Unextruded	Extruded	Unextruded	Extruded		
DM										
a (% of DM)	31.1^b^	45.5^a^	30.6^b^	43.0^a^	25.2^bc^	41.8^a^	20.7^c^	31.2^b^	1.94	<0.001
b (% of DM)	52.4^a^	32.8^c^	51.0^a^	39.0^bc^	56.2^a^	41.3^b^	54.4^a^	41.7^b^	1.82	<0.001
c (%/h)	6.45^ab^	6.15^ab^	5.63^bc^	7.66^a^	4.26^c^	6.29^ab^	5.40^bc^	6.28^ab^	0.26	0.040
ED (% of DM)	60.3^bc^	64.0^ab^	57.3^cd^	66.7^a^	50.5^ef^	64.9^a^	48.7^f^	54.3^de^	1.38	<0.001
CP										
a (% of CP)	31.7^b^	41.1^a^	40.5^a^	47.9^a^	19.6^c^	42.8^a^	31.7^b^	33.0^b^	1.89	<0.001
b (% of CP)	63.8^a^	51.3^bc^	53.6^ab^	40.6^c^	63.4^a^	48.9^bc^	56.5^ab^	54.1^ab^	1.82	0.007
c (%/h)	4.96^ab^	4.82^ab^	3.03^b^	4.24^ab^	5.98^a^	3.86^ab^	4.30^ab^	5.45^ab^	0.29	0.244
ED (% of CP)	63.6^ab^	66.3^a^	60.8^bc^	65.6^a^	53.7^d^	63.6^ab^	57.1^cd^	60.8^bc^	0.91	<0.001
NDF										
a (% of NDF)	11.1^a^	10.1^abc^	6.76^c^	9.48^abc^	7.42^bc^	9.18^abc^	7.92^abc^	10.4^ab^	0.43	0.080
b (% of NDF)	40.8^ab^	42.3^a^	39.2^ab^	43.1^a^	39.1^ab^	43.5^a^	39.9^ab^	35.2^b^	0.77	0.105
c (%/h)	2.24^c^	2.97^bc^	3.60^ab^	3.67^ab^	4.09^a^	3.21^abc^	2.84^bc^	2.95^bc^	0.14	0.015
ED (% of NDF)	23.4^bc^	25.7^abc^	23.0^bc^	27.3^a^	24.7^abc^	25.9^ab^	22.1^c^	23.3^bc^	0.48	0.056
ADF										
a (% of ADF)	8.41	8.80	5.99	7.17	4.94	8.13	7.67	8.42	0.53	0.652
b (% of ADF)	36.4^b^	36.0^b^	38.4^ab^	41.4^ab^	48.0^a^	42.0^ab^	40.1^ab^	41.2^ab^	1.19	0.248
c (%/h)	2.69	3.61	3.18	3.59	2.51	3.09	2.67	2.42	0.18	0.637
ED (% of ADF)	20.7	23.8	20.2	23.6	20.7	24.0	21.0	21.2	0.45	0.117

1) a, soluble fraction; b, slowly degradable fraction; c, slowly degradation rate; ED, effective degradability.

a–f Superscripted letters indicate significant differences based on Tukey’s tests, with significance defined as p<0.05.

SEM, standard errors of the mean; DM, dry matter; CP, crude protein; NDF, neutral detergent fiber; ADF, acid detergent fiber.

**Table 4:** Effects of feeding extruded compound feed on growth performance of fattening cattle

Items	Treatments^1)^		SEM	Day			SEM	p-value		
		
	CON	EXP		0–30 d	31–60 d	0–60 d		Treatment	Day	Treatment×Day
ADFI (kg)	16.0	17.2	0.08	15.4	17.8	16.6	0.10	<0.001	<0.001	<0.001
ADG (kg)	1.51	1.72	0.02	1.53	1.69	1.61	0.03	<0.001	0.002	0.311
F/G	10.6	10.0	0.09	10.1	10.6	10.3	0.11	0.008	0.026	0.968

1) CON, the corn-soybean meal-peanut vine-corn silage-based diet (used as the control diet); EXP, the extruded compound feed (substituted equal amount of feed ingredients).

SEM, standard errors of the mean; ADFI, average daily feed intake; ADG, average daily gain; F/G, feed intake/body weight gain.

**Table 5:** Effects of feeding extruded compound feed on economic benefits of fattening cattle

Items	Treatments^1)^	

	CON	EXP
Feed unit price (USD/kg)	0.23	0.25
ADFI (kg/head)	16.0	17.2
Total 60 d feed intake (kg/head)	960	1,032
Total 60 d feed cost (USD/head)	221	258
ADG (kg/head)	1.51	1.72
Total 60 d weight gain (kg/head)	90.6	103.2
Unit price of fattening cattle (USD/kg)	3.50	3.50
Weight gain income (USD/head)	317.1	361.2
Feeding profit (USD/head)	96.1	103.2

1) CON, the corn-soybean meal-peanut vine-corn silage-based diet (used as the control diet); EXP, the extruded compound feed (substituted equal amount of feed ingredients).

USD, US dollars; ADFI, average daily feed intake; ADG, average daily gain.

**Table 6:** Effects of feeding extruded compound feed on nutrient apparent digestibility of fattening cattle

Items	Treatments^1)^		SEM	p-value

	CON	EXP		
DM	79.0	83.0	0.72	0.002
CP	78.7	87.0	1.43	<0.001
EE	90.4	89.7	0.68	0.669
NDF	60.5	67.0	1.37	0.013
ADF	56.7	62.5	1.69	0.083

1) CON, the corn-soybean meal-peanut vine-corn silage-based diet (used as the control diet); EXP, the extruded compound feed (substituted equal amount of feed ingredients).

SEM, standard errors of the mean; DM, dry matter; CP, crude protein; EE, ether extract; NDF, neutral detergent fiber; ADF, acid detergent fiber.

**Table 7:** Effects of feeding extruded compound feed on rumen fermentation parameters of fattening cattle

Items	Treatments^1)^		SEM	p-value

	CON	EXP		
pH	6.86	6.48	0.05	<0.001
Ammonia nitrogen (mM)	24.2	27.7	0.41	<0.001
Acetate (mM)	56.2	68.0	1.86	<0.001
Propionate (mM)	19.9	27.9	0.87	<0.001
Butyrate (mM)	16.1	18.8	0.76	0.071
Pentanoate (mM)	1.43	1.40	0.03	0.609
Isovalerate (mM)	1.36	1.44	0.05	0.403
Acetate-to-propionate	2.86	2.45	0.09	<0.001
Total volatile fatty acids (mM)	95.0	117.6	3.00	0.018

1) CON, the corn-soybean meal-peanut vine-corn silage-based diet (used as the control diet); EXP, the extruded compound feed (substituted equal amount of feed ingredients).

SEM, standard errors of the mean.

**Table 8:** Effects of feeding extruded compound feed on rumen bacterial diversity of fattening cattle

Items	Treatments^1)^		SEM	p-value

	CON	EXP		
Coverage	0.99	0.99	0.0003	0.465
Sobs	1,184.5	1,300.2	62.8	0.382
Shannon	5.06	5.08	0.13	0.939
Simpson	0.02	0.04	0.01	0.438
Ace	1,367.9	1,507.4	65.8	0.312
Chao1	1,373.5	1,524.2	69.9	0.302

1) CON, the corn-soybean meal-peanut vine-corn silage-based diet (used as the control diet); EXP, the extruded compound feed (substituted equal amount of feed ingredients).

SEM, standard errors of the mean.

## Data Availability

Upon reasonable request, the datasets of this study can be available from the corresponding author.
